# Prediction and Classification the Risk of Stroke Patients Using Machine Learning Techniques: A Retrospective Cross‐Sectional Study

**DOI:** 10.1002/hsr2.71419

**Published:** 2025-10-28

**Authors:** Ghasem Alizadeh‐dizaj, Shiva Khoshsirat

**Affiliations:** ^1^ School of Management and Medical Informatics Tabriz University of Medical Sciences Tabriz Iran

**Keywords:** machine learning techniques, prediction, risk classification, stroke

## Abstract

**Background:**

Stroke is one of the most common causes of death and neurological disabilities in all societies. The use of machine learning techniques to create predictive models is very helpful in identifying people at risk to reduce the complications of the disease. The purpose of this study was to investigate the performance of machine learning algorithms and predict the risk of stroke in suspected stroke patients using decision tree based on the risk factors that affect it.

**Methods:**

The study analyzed medical records of 1184 stroke‐suspected patients presenting at an Emergency Department using machine learning algorithms. Attributes such as age, primary diagnoses, gender, blood pressure, smoking, diabetes, and other relevant factors were considered. The data set was preprocessed to handle missing and incompatible data. Algorithms like Naïve Bayes, Neural Network, kNN, SVM, and Classification Tree were applied, with a training‐test data split of 70–30 using K‐fold Cross Validation.

**Results:**

Among the machine learning algorithms used, kNN demonstrated the highest accuracy (97.30%), sensitivity (98.75%), specificity (98.72%), and F1 criteria (98.66%) in predicting stroke severity. Physical inactivity, high cholesterol, cardiovascular disease, history of transient ischemic attack, and high blood pressure emerged as the most influential risk factors for stroke severity. Decision Tree analysis provided valuable insights into the relationship between risk factors and stroke severity.

**Conclusion:**

machine learning techniques proved effective in identifying risk factors and predicting stroke severity, showing promise in enhancing stroke management strategies. The study highlighted the importance of physical inactivity and other key risk factors in stroke prediction. Consistency in risk factor importance across studies suggests common underlying factors, while acknowledging variations based on geographic and lifestyle factors.

## 1 Introduction

Stroke or Cerebrovascular Accident (CVA) is the third leading cause of death in the United States and is the most common neurological disorder in adults worldwide [1, 2, 3]. Annually, 750,000 new cases of stroke occur in the United States, resulting in approximately 150,000 deaths [4, 5]. It ranks sixth in the global disease burden and is projected to rise to fourth place by 2025 [6, 7, 8]. The annual incidence of stroke is estimated at 15 million worldwide, with only one‐third experiencing full recovery; one‐third succumb to the condition, and one‐third face impotence [9, 10]. In Iran, 139 out of every 100,000 individuals experience a first‐time stroke each year. The incidence of stroke in individuals over 45 years old exceeds 500 cases per 100,000 people, significantly higher than in developed nations [11, 12]. Previous research indicates that the risk of stroke doubles with each decade after the age of 55 [13]. Consequently, the economic impact of cerebrovascular diseases, including hospitalization expenses, rehabilitation costs, and managing stroke‐related complications, is substantial and contributes significantly to a country's healthcare expenditures [14, 15]. Moreover, developing countries like Iran are rapidly aging due to improved public health, necessitating comprehensive healthcare services [16, 17]. Despite its lethality, stroke is preventable, and efforts to reduce its occurrence for public health improvement are crucial, with awareness of its risk factors being the primary strategy for achieving this goal [18, 19]. Previous studies consistently highlight that identifying and managing risk factors can lower the incidence of this disease [20, 21]. One way to reduce the cost of bed occupancy and identify individuals at risk is to develop a classification system, a crucial aspect of clinical medicine that aids in diagnosing and treating high‐risk patients [22]. In the last 15 years, several classification schemes have been proposed to assess stroke risk, but they have shown limited predictive value [23]. Peeters et al. stress the importance of having risk classification models [24]. However, current risk prediction models face several limitations. First, stroke is a multifactorial disease with complex interactions between genetic, lifestyle, and clinical factors (e.g., hypertension, diabetes, atrial fibrillation), making accurate risk stratification difficult [24]. Existing models, such as the Framingham Stroke Risk Profile (FSRP) and QStroke, often rely on traditional statistical methods (e.g., logistic regression) and have shown moderate predictive performance, with AUCs typically ranging between 0.70 and 0.80 in validation studies [25, 26]. While some machine learning‐based models achieve higher AUCs (e.g., 0.85–0.90 in select cohorts), their generalizability is limited due to heterogeneous populations and varying risk factor definitions [27]. Additionally, sensitivity and specificity trade‐offs remain a challenge; for instance, models prioritizing sensitivity to capture high‐risk cases may suffer from lower specificity, leading to unnecessary interventions [28]. Another critical issue is the dynamic nature of stroke risk. Most models provide static predictions rather than real‐time updates based on evolving patient data, limiting their clinical utility [29]. Furthermore, many models are derived from Western populations, raising concerns about their applicability to diverse ethnic groups, such as those in Iran, where stroke epidemiology and risk factor profiles may differ [30].

Given these challenges, machine learning techniques offer a promising alternative by uncovering hidden patterns in large, complex datasets. Unlike traditional methods, machine learning algorithms (e.g., random forests, neural networks) can handle nonlinear relationships and high‐dimensional data, potentially improving prediction accuracy [31, 32]. Recent studies demonstrate that machine learning approaches can achieve AUCs exceeding 0.85, with sensitivity and specificity above 80% in some cases, though further validation is needed [33, 34]. These techniques also enable personalized risk assessment by integrating diverse data sources (e.g., electronic health records, imaging, and genetic data), facilitating early intervention and resource optimization [35]. This study aims to identify signs and risk factors for stroke disease through machine learning techniques and to establish a model for classifying and predicting the severity of stroke disease.

## 2 Methods

Medical records of all patients entering the Emergency department of Imam Reza Medical Center with a suspected stroke diagnosis between November and February of 2023 comprised the research population. Sampling was not conducted, and all 1184 cases were included in the study. Patients admitted for reasons other than disturbances of consciousness, dysarthria, facial paralysis, limb numbness, and half‐body weakness were excluded. Attributes were identified after reviewing relevant literature and consulting with seven CVA clinical experts. An Excel file was created for data collection and recording, including age, primary diagnoses, gender, blood pressure (HTN), smoking, diabetes (DM), high cholesterol, physical inactivity, carotid or other artery diseases, transient ischemic attacks, atrial fibrillation, other heart diseases, certain blood disorders, alcohol consumption, illegal drug use, pre‐stroke history, family history of stroke, and stroke risk level. The preprocessing phase involved meticulous data cleaning to handle missing values and outliers, along with feature scaling to standardize variable magnitudes. Data was collected using the Excel‐Data mining software Add‐Ins tool, and missing, noisy, and incompatible data were appropriately addressed. Missing data in 16 records were replaced with the mode of the same attribute, while five incompatible data records were replaced with the mode of the same attribute. Seven incompatible data points for age were replaced with the average value for that attribute. Of the 18 attributes in the study, age was continuous, while the rest were discrete. The eighteenth attribute, with values of Low, Moderate, High, and Very High, was designated as the target attribute indicating stroke risk, as determined by clinical experts in the field. This attribute was considered by the clinical experts in this area as bellows.

If a patient with the aforementioned complaints visits the emergency department (ED) and stays less than 6 h, the categorization would be “low”; staying in the ED for 6–18 h would be considered “moderate”. If the patient stays over 18 h in the ED, gets admitted to the neurology ward, or is referred to another facility for further care, the categorization would be “High”. If, following initial interventions, the patient is admitted to the neurology Intensive Care Unit (ICU) or passes away, the categorization would be “Very High”. This approach balances model simplicity with predictive power, aligning with clinical risk assessment tools like the ABCD2 score [36] and CHA2DS2‐VASc [37]. The target variable (stroke risk: Low/Moderate/High/Very High) was defined based on established clinical triage protocols and expert consensus.

In this study, the most recent versions of Orange (Version 2.7.8), Naïve Bayes, Neural Network, kNN, SVM, and Classification Tree machine learning algorithms were utilized. Orange (Version 2.7.8) was chosen for its user‐friendly interface and comprehensive functionalities for data exploration, visualization, and machine learning. The selection of specific machine learning algorithms in this study, was based on the diverse strengths and capabilities of each model in analyzing complex datasets related to stroke risk factors. Naïve Bayes was included due to its simplicity, speed, and efficiency in handling categorical datasets. Neural Network, known for its ability to capture complex nonlinear relationships, was selected to uncover intricate patterns in stroke prediction data. kNN was chosen for its power in identifying patterns among similar patient profiles based on nearest neighbor relationships. SVM, ideal for high‐dimensional datasets with nonlinear boundaries, was included for its capability to handle complex data structures in stroke risk assessment. The Classification Tree algorithm, with its interpretability and ability to capture hierarchical interactions among variables, was used to reveal decision rules for predicting stroke outcomes.

70% of the data (829 samples) was used for training the system, while 30% (355 samples) was used as test data. The K‐fold Cross Validation model with a value of 5 was employed in this study. This split helps to ensure that the model is trained on a sufficiently large data set to learn the underlying patterns and relationships in the data while retaining enough data for evaluation to ensure the model generalizes well to unseen data. On the other hand, a value of *K* = 5 (in *K*‐fold cross‐validation technique) was used because it strikes a balance between computational efficiency and reliable estimation of the model's performance. With five folds, the data is divided into five subsets, and the model is trained and tested five times, each time using a different subset as the validation set. This approach provides a more robust estimate of the model's performance compared to a simple train‐test split, as it helps reduce the variability in model evaluation that may result from using a single test set. To mitigate overfitting and enhance clinical applicability, we performed feature selection using Orange's Rank widget (based on information gain) and retained the top 17 most predictive variables. We have rigorously validated our models against overfitting using cross‐validation, feature selection, and regularization, confirming their robust performance on unseen test data. These additions strengthen the study's reliability and provide a clear pathway for further research.

## 3 Results

Out of 1184 samples, 165 (13.9%) were categorized as low, 228 (19.3%) as moderate, 561 (47.4%) as high, and 230 (19.4%) as very high. The age range of the samples in the data set was between 28 and 100 years (Mean = 68.24). Among the total samples, 530 cases (44.8%) were female, and 654 (55.2%) were male. The evaluation criteria in this study included accuracy, sensitivity, specificity, precision, recall, and F‐measure, calculated based on the Confusion Matrix, as reported in Table 1.

**Table 1:** Results from the implementation of algorithms.

**Validation method**						
**Method:** Cross‐validation						
**Folds:** 5						
**Target class:** High						
**Data**						
**Examples:** 1184						
**Attributes:** 17 (Age, Dx, Gender, HTN, Smoking, DM, High_Chol, PH_Inactivity, CAD, TIA, Afib, Other_HD, CBD, Alc, illegal_drug, Pstroke, FH)						
**Class:** Risk_level						
**Results**						
	**CA**	**Sens**	**Spec**	**F1**	**Prec**	**Recall**
**Naive Bayes**	0.7373	0.8004	0.7769	0.7815	0.7636	0.8004
**Neural Network**	0.9375	0.9608	0.9856	0.9720	0.9836	0.9608
**kNN**	0.9730	0.9875	0.9872	0.9866	0.9858	0.9875
**SVM**	0.7508	0.8217	0.7897	0.7997	0.7787	0.8217
**Classification Tree**	0.9552	0.9715	0.9711	0.9698	0.9680	0.9715

**Figure 1:**
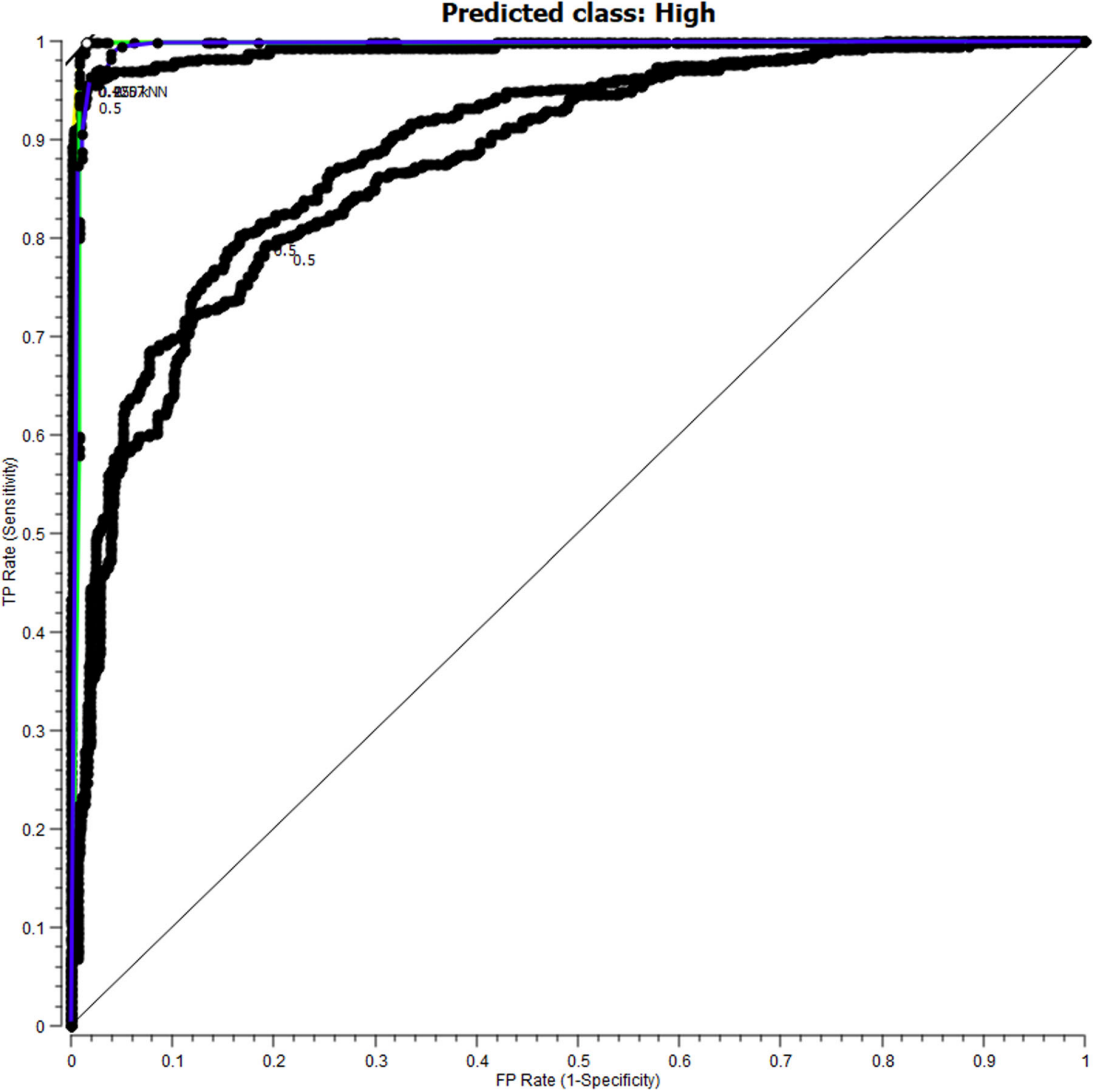
ROC curves for algorithms implemented with predicted class High.

According to Table 1. The k‐Nearest Neighbor (kNN) has the best performance compared to other algorithms, with an accuracy of 97.30%, a sensitivity of 98.75%, a specificity of 98.72%, F1 criteria of 98.66%, a precision of 98.58%, and its retrieval was 98.75%. Also, according to the results obtained from the evaluation of the algorithms, the weakest performance was for the Naive Bayes algorithm. Figure 1. Shows the ROC curves for the implemented algorithms with the predicted class high.

**Figure 2:**
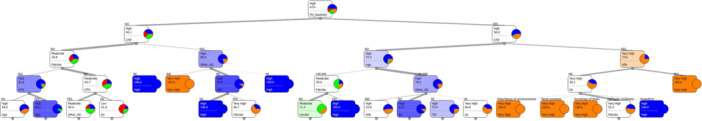
Decision tree for hazard classification in patients with stroke referral to the emergency department.

To understand the results of the study and obtain predictive rules decision tree was used for constructing the stroke risk classification model (Figure 2).

The decision tree had five levels (Max Tree Depth = 5) and consisted of 127 leaves and 225 nodes. The rules are:

- If a person with a physical activity, a coronary artery disease, a history of heart failure or a history of a heart attack, the likelihood of being in the “high” class is 100%. In other words, all people with these conditions are likely to be stroke.
- If a person with physical activity, coronary artery disease, and ischemic heart disease has a high probability of being in “very high” class is 100%.
- If a person with a low physical activity and having a coronary artery disease without atrial fibrillation and with one of the primary complaints of disturbances of consciousness, facial paralysis, numbness of limbs the likelihood of being high in the class is 100%.
- If a person with a low physical activity, without coronary artery disease, older than 85 years of age has a history of stroke, the likelihood of being in the “high” class is 100%.
- If a person with a physical activity, a coronary artery disease, a history of heart failure or a history of a heart attack, the likelihood of being in the “high” class is 100%.
- If a person with appropriate physical activity, no coronary artery disease and a history of previous stroke and blood pressure, the likelihood of being in the “high” class is 61.8%.
- If a person with a suitable physical activity, without coronary artery disease, with a history of previous stroke and blood pressure, is likely to be in the “high” class, 88.2%.
- If a person with appropriate physical activity, no coronary artery disease and a history of previous stroke and blood pressure, the likelihood of being in the “high” class is 61.8%.
- According to Table 2. The PH_Inactivity (physical activity) attribute had the highest value of Information Gain (IG) than the other attributes and was placed in the root node. This was done repeatedly and continuously to determine the nodes of the next tree level.

**Table 2:** Results of calculation of Information Gain for each attribute.

	Attribute	Inf. gain
1	PH_Inactivity	0.2761
2	High_Chol	0.2434
3	CAD	0.2279
4	TIA	0.2017
5	Afib	0.1823
6	Other_HD	0.1141
7	Pstroke	0.1123
8	HTN	0.0842
9	Age	0.0814
10	CBD	0.0545
11	FH	0.0482
12	Smoking	0.0439
13	DM	0.0415
14	Dx	0.0338
15	illegal_drug	0.0295
16	Alc	0.0166
17	Gender	0.0044

## 4 Discussion

Early diagnosis and rapid intervention in stroke are essential and reduce mortality and, more importantly, reduce complications among patients. But an appropriate estimate of the risk in patients, such as the diagnosis of some stroke symptoms, is difficult and complicated. There are currently no accepted procedures for classifying patients, and existing studies do not classify patients based on their risk factors, rather they classify them according to clinical symptoms or clinical findings.

After reviewing the studies in the stroke area and with the opinion of clinical experts, 18 attributes were selected for data collection, of which 17 were predictive attributes and one was the target attribute. Mehdi Pour et al. of Used 22 attributes to predict stroke in their study, of which 14 were consistent with the attributes of our study. Also, Arsalan et al., In a study aimed at predicting ischemic stroke used 18 attributes of which three attributes were similar to our study [38]. In another study by Amini et al. With the aim of predicting and controlling stroke by machine learning techniques, researchers reported that 50 of the risk factors affecting stroke were significantly higher than the variables of our study [39]. Our model's risk factors align with regional epidemiological studies (e.g., hypertension, AFib), but differences emerge due to hospital‐based versus population‐level data (e.g., stronger carotid disease association in our cohort). These variations highlight the importance of context‐specific models while epidemiological studies identify population risks, clinical models like ours optimize individualized in‐hospital prediction.

The most effective attribute in the study was physical inactivity. High cholesterol, cardiovascular disease, history of transient ischemic attack, history of previous stroke and high blood pressure status were the five most effective risk factors in our study. In this regard, the study of Mehdi Pour et al. Showed that diabetes mellitus was the highest risk factor for stroke, which contradicted this study [40]. Migraines, transient ischemic attacks, anemia, and myocardial infarction were also important respectively. Aigner et al. In a study aimed at assessing the contribution of potentially cardiovascular risk factors to stroke burden in young adults in Germany, after examining eight risk factors including hypertension, hyperlipidemia, diabetes mellitus, coronary heart disease, smoking, intake Excessive alcohol, low physical activity, and obesity, reported that low physical activity and high blood pressure were the most important risk factors associated with stroke, the results were consistent with the results of our study [41]. The systematic review and meta‐analysis by Wang et al. on the risk factors of stroke in the Chinese population showed that blood pressure, diabetes mellitus, family history of stroke, hyperlipidemia, obesity and smoking were the most important risk factors for stroke Which was consistent with the results of the present study [42], In addition, there was a smoker attribute in our study, but had less importance than five factors in the study. It seems that the lack of proper documentation and incomplete records could have decreased the effect of smoking on stroke.

Also, Qawasmeh et al., in an epidemiological study of stroke risk factors, reported that blood pressure, ischemic heart disease and diabetes were the most important stroke risk factors that were consistent with our study [43]. According to the above, it seems the risk factors of stroke are similar in various studies, but due to the factors such as climate and lifestyle, the importance of risk factors in different countries is not the same. Also, due to some limitation such as time period for data collection and research population which was limited to one medical center and the physician' documentation approach, it seems that all of these factors can be effective on the importance of risk factors for stroke.

This study showed that the kNN algorithm with a slight difference to the Classification Tree algorithm has the best performance among the algorithms used. Govindarajan et al., In a study aimed at providing a stroke predictive model using machine learning classification methods, using three decision tree, Naive Bayes, and Neural Network algorithms, reported that the Neural Network algorithm performed the best performance [22]. In this regard, the study by Amini et al. with the aim of predicting and controlling of stroke by machine learning techniques, using two decision‐tree C4.5 and K nearest neighbor, reported that C4.5 Decision Tree algorithm with 95.42 percent accuracy was slightly better than the K‐Neighbor algorithm with an accuracy of 94.18%, which is consistent with our study [39]. Also, in another study by Almadani et al. With the aim of stroke prediction using the machine learning stratification which used three C4.5, JRip and Neural Network algorithms, the results showed that the C4.5 algorithm with 95.25%, had the most accuracy compared to the two others [44]. Arslan et al., in their study aimed at predicting stroke with various machine learning algorithms using Support Vector Machine algorithms, Stochastic Gradient Boosting and Penalized Logistic Regression, reported that Support Vector Machine algorithm with 97.89% compared to other algorithms had the best performance in terms of accuracy, which contradicts our research results [38].

The performance of the mentioned algorithms can be related to various factors, such as the nature of the data collected, the number and type of attributes selected, the number of records collected, the pre‐processing of the data set, methods used to evaluate the performance of algorithms, the frequency and manner of dividing the data set into training data and test data, and other factors.

By comparing the results and performance of the algorithms used in our study with other studies, the superiority of our study is determined in several ways:

**High Accuracy and Performance:** The kNN algorithm in our study achieved a high accuracy of 97.30%, demonstrating superior performance in predicting stroke severity compared to other algorithms used in the study. This high level of accuracy is crucial for effective risk prediction and management.

**Identification of Key Risk Factors:** our study highlighted specific risk factors such as physical inactivity, high cholesterol, cardiovascular disease, and history of transient ischemic attack as significant contributors to stroke severity. By pinpointing these key factors, the study provides valuable insights for improving stroke management strategies.

**Consistency with Existing Literature:** While there are variations in the importance of certain risk factors across studies, our study's findings align with previous research on stroke risk factors. The study's emphasis on physical inactivity and other common risk factors adds to the existing body of knowledge of stroke prevention and management.

**Effective Use of Machine Learning Techniques:** By leveraging machine learning algorithms, our study demonstrates the potential of these techniques in identifying risk factors and predicting stroke severity. The application of algorithms like kNN showcases the effectiveness of machine learning in healthcare settings for analyzing complex datasets and making accurate predictions.

This study presents a clinically validated stroke prediction model with 97.3% accuracy, demonstrating significant potential for both healthcare implementation and public health policy. For clinical practice, the model enables real‐time risk stratification in emergency settings, improving triage efficiency and treatment prioritization. Simultaneously, its identification of high‐impact modifiable risk factors provides evidence‐based targets for regional stroke prevention programs. While particularly relevant for Iranian population, the model's robust validation framework addresses potential overfitting concerns, ensuring reliable performance across different healthcare settings. The findings support two key implementation pathways: integration with hospital EHR systems for bedside decision support, and application in public health planning through regional risk mapping and targeted prevention strategies. These complementary applications bridge the gap between individual patient care and population health management, offering a comprehensive approach to stroke risk reduction.

Overall, our study's use of machine learning algorithms, the identification of key risk factors, high accuracy in predicting stroke severity, and alignment with existing literature on stroke risk factors collectively contribute to its significance and potential impact on enhancing stroke management practices. While the model shows robust accuracy, we explicitly address overfitting risks through cross‐validation and regularization, as noted in limitations. These refinements strengthen its translational potential for both bedside and population‐level use.

While our study offers valuable insights, several limitations should be considered. These include the relatively small sample size, which may limit the generalizability of our findings, and the potential impact of data quality issues and missing values on the performance of our algorithms. The selection of features and algorithms in our analysis could also influence the predictive capabilities of our models. Additionally, the lack of external validation and clinical interpretation of our results highlights the need for further research to validate and refine our predictive models for stroke risk assessment. Addressing these limitations will be essential for enhancing the reliability and applicability of our findings in clinical practice and healthcare decision‐making.

## 5 Conclusion

Machine Learning Techniques proved effective in identifying risk factors and predicting stroke severity, showing promise in enhancing stroke management strategies. While gender showed lower importance compared to other factors, factors like high blood pressure, ischemic heart disease, and diabetes emerged as significant contributors to stroke risk, consistent with existing literature. The study found the k‐Nearest Neighbor algorithm to perform best among the algorithms tested, with high accuracy rates. The results emphasize the importance of factors such as physical inactivity and high cholesterol in predicting stroke severity. While variations in algorithm performance exist across studies, the study underscores the clinical relevance of machine learning techniques in improving stroke risk assessment and management. This scientific added value extends beyond traditional risk assessment methods, offering a more data‐driven and precise approach to identifying and mitigating stroke risk factors.

Looking ahead, our study opens up avenues for further research and validation of our findings in larger population cohorts to strengthen the reliability and generalizability of our predictive models. By continuing to explore the use of machine learning techniques in healthcare, particularly in the context of stroke prediction, we can advance the development of more accurate and tailored strategies for early intervention and prevention efforts. Ultimately, our research contributes to the ongoing pursuit of improving patient outcomes and reducing the burden of stroke through innovative and evidence‐based approaches.

## Author Contributions

**Conceptualization, Data collection** and **analysis, Writing original draft:** Ghasem Alizadeh‐dizaj and Shiva Khoshsirat. All authors have read and approved the final version of the manuscript. Corrisponding author, Ghasem Alizadeh‐dizaj had full access to all of the data in this study and takes complete responsibility for the integrity of the data and the accuracy of the data analysis.

## Ethics Statement

This study was approved by the Research Ethics Committee of Tabriz University of Medical Sciences with approval code IR. TBZMED. REC.1396.714.

## Consent

Before data collection began, participants were provided with detailed information about the study's objectives, procedures, and privacy practices. Informed consent was obtained from the participant to ensure transparency and ethical behavior. Confidentiality protocols were strictly followed to maintain the anonymity and privacy of the participants. Data were anonymized, securely stored, and accessible only to the research team. Participation in the study was voluntary and participants had the right to withdraw at any stage of the research process without any consequences.

## Conflicts of Interest

The authors declare no conflicts of interest.

## Transparency Statement

The lead author Ghasem Alizadeh‐dizaj affirms that this manuscript is an honest, accurate, and transparent account of the study being reported; that no important aspects of the study have been omitted; and that any discrepancies from the study as planned (and, if relevant, registered) have been explained.

## Data Availability

All data used and analyzed during the current study are available from the corresponding author upon reasonable request.
